# Diaqua­bis­(2-oxo-2*H*-chromene-3-carboxyl­ato-κ^2^ *O* ^2^,*O* ^3^)manganese(II)

**DOI:** 10.1107/S1600536811016667

**Published:** 2011-05-07

**Authors:** Yue Cui, Qian Gao, Huan-Huan Wang, Lin Wang, Ya-Bo Xie

**Affiliations:** a College of Environmental and Energy Engineering, Beijing University of Technology, Beijing 100124, People’s Republic of China

## Abstract

In the title compound, [Mn(C_10_H_5_O_4_)_2_(H_2_O)_2_], the Mn^II^ atom lies on a crystallographic inversion center and is six-coordinated by two O atoms from water mol­ecules in the axial positions and four O atoms from two deprotonated coumarin-3-carb­oxy­lic acid ligands in the equatorial plane. The overall coordination geometry is slightly distorted octa­hedral. The Mn—O bond distances vary between 2.0931 (12) and 2.2315 (13) Å. O—H⋯O hydrogen bonds between the H atoms of coordinated water mol­ecules and the O atoms of the carboxyl­ate groups link the complex mol­ecules into two-dimensional layers parallel to the *ab* plane.

## Related literature

For background to topological networks, see: Hu *et al.* (2010 ▶). For applications of manganese(II) complexes, see: Hazra *et al.* (2011 ▶), Kuschel *et al.* (2010 ▶); Yang *et al.* (2010 ▶). For related structures, see: Gao *et al.* (2010 ▶).
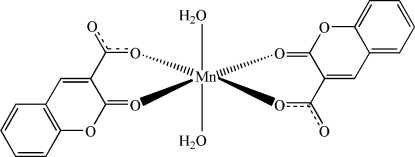


## Experimental

### 

#### Crystal data

[Mn(C_10_H_5_O_4_)_2_(H_2_O)_2_]
*M* *_r_* = 469.25
Triclinic, 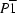

*a* = 6.7036 (13) Å
*b* = 6.9797 (14) Å
*c* = 10.424 (2) Å
α = 93.28 (3)°
β = 90.67 (3)°
γ = 113.47 (3)°
*V* = 446.33 (15) Å^3^
*Z* = 1
Mo *K*α radiation
μ = 0.80 mm^−1^
*T* = 293 K
0.20 × 0.20 × 0.15 mm

#### Data collection

Bruker APEXII CCD diffractometer
Absorption correction: multi-scan (*SADABS*; Bruker, 2008 ▶) *T*_min_ = 0.852, *T*_max_ = 0.887
2811 measured reflections
2021 independent reflections
1905 reflections with *I* > 2σ(*I*)
*R*_int_ = 0.009

#### Refinement

*R*[*F* ^2^ > 2σ(*F* ^2^)] = 0.027
*wR*(*F* ^2^) = 0.070
*S* = 1.07
2021 reflections
146 parameters
H atoms treated by a mixture of independent and constrained refinement
Δρ_max_ = 0.27 e Å^−3^
Δρ_min_ = −0.25 e Å^−3^

### 

Data collection: *APEX2* (Bruker, 2008 ▶); cell refinement: *SAINT* (Bruker, 2008 ▶); data reduction: *SAINT*; program(s) used to solve structure: *SHELXS97* (Sheldrick, 2008 ▶); program(s) used to refine structure: *SHELXL97* (Sheldrick, 2008 ▶); molecular graphics: *SHELXTL* (Sheldrick, 2008 ▶); software used to prepare material for publication: *SHELXL97*.

## Supplementary Material

Crystal structure: contains datablocks global, I. DOI: 10.1107/S1600536811016667/sj5131sup1.cif

Structure factors: contains datablocks I. DOI: 10.1107/S1600536811016667/sj5131Isup2.hkl

Additional supplementary materials:  crystallographic information; 3D view; checkCIF report

## Figures and Tables

**Table 1:** Hydrogen-bond geometry (Å, °)

*D*—H⋯*A*	*D*—H	H⋯*A*	*D*⋯*A*	*D*—H⋯*A*
O1*W*—H1*WA*⋯O4^i^	0.82	1.89	2.7113 (17)	178
O1*W*—H1*WB*⋯O4^ii^	0.82 (3)	1.95 (3)	2.755 (2)	167 (2)

Symmetry codes: (i) 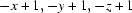; (ii) 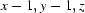.

**Fig. 1.**
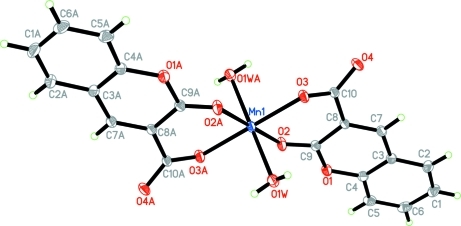
The molecular structure of the title compound with displacement ellipsoids drawn at the 50% probability level for non-hydrogen atoms, hydrogen atoms are shown as small circles of arbitrary radius. [Symmetry code: A = -x, -y + 1, -z + 1].

**Fig. 2.**
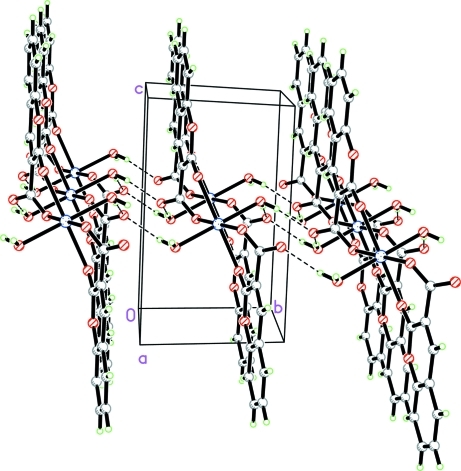
Partial packing view of title compound, showing the formation of network built from hydrogen bonds.

## supplementary crystallographic information

### Comment

In the past decades, numerous papers dealing with manganese(II) complexes have been published due to their fascinating structural diversity (Hu *et al.*, 2010) and potential applications in the areas of catalysis (Kuschel *et al.*, 2010), gas adsorption (Hazra *et al.*, 2011) and magnetism (Yang *et al.*, 2010). Herein, we report the synthesis and crystal structure of a new mononuclear manganese complex coordinated by coumarin-3-carboxylic acid.

In the title compound, [Mn(C_10_H_5_O_4_)_2_(H_2_O)_2_], each manganese(II) atom lies on a crystallographic inversion center and is six-coordinated by two O atoms from water molecules in the axial positions and four O atoms from two deprotonated coumarin-3-carboxylic acid ligands in the equatorial plane to exhibits a slightly distorted octahedral geometry. Angles around the Mn(II) atom vary between 82.88 (5)° and 97.12 (5)°. The Mn—O bond distances between the Mn(II) atom and the O atoms vary between 2.0931 (12) and 2.2315 (13) Å, all of which are comparable to those reported for other manganese-oxygen donor complexes (*e.g.* Gao *et al.*, 2010). The (C1/C2/C3/C4/C5/C6) and (C4/C3/C7/C8/C9/O1) rings are almost coplanar with a dihedral angle of 1.847 (6)° between them. The dihedral angle between the C10/C8/C9/O2 and O2/Mn/O3 planes is 25.803 (7)°. O—H···O hydrogen bonds between the hydrogen atoms of the coordinated water molecules and the O atoms of the carboxyl groups [O(1 W)—H(1 W A)···O(4), 2.711 (2) Å, O(1 W)—H(1WB)···O(4), 2.755 (2) Å] join the complexes into two-dimensional layers parallel the *ab* plane (Table 1, Fig. 2).

### Experimental

The title complex was synthesized by carefully layering a solution of MnSO_4_.H_2_O (16.9 mg, 0.1 mmol) in ethanol (10 ml) on top of a solution of coumarin-3-carboxylic acid (19.0 mg, 0.1 mmol) and LiOH (8.4 mg, 0.2 mmol) in H_2_O (10 ml) in a test-tube. After about one month at room temperature, yellow block-shaped single crystals suitable for X-ray investigation appeared at the boundary between the ethanol solution and the water layer with a yield of 25% (12.1 mg). FT—IR (KBr, cm^-1^): 788, 1028, 1183, 1285, 1388, 1457, 1585, 1662, 3214.

### Refinement

Carbon H atoms were placed geometrically (C—H = 0.93 Å) and treated as riding with U_iso(H)_ = 1.2U_eq_(C). Water H atoms were located in  a difference Fourier map. One was treated as riding in the subsequent refinement atoms, with O—H = 0.85 Å, the coordinates of the other were refined freely.  For both i>U_iso_(H) = 1.5U_eq_(O).

### Crystal data

**Table:**

[Mn(C_10_H_5_O_4_)_2_(H_2_O)_2_]	*Z* = 1
*M**_r_* = 469.25	*F*(000) = 239
Triclinic, *P*1	*D*_x_ = 1.746 Mg m^−^^3^
Hall symbol: -P 1	Mo *K*α radiation, λ = 0.71073 Å
*a* = 6.7036 (13) Å	Cell parameters from 1947 reflections
*b* = 6.9797 (14) Å	θ = 2.0–28.3°
*c* = 10.424 (2) Å	µ = 0.80 mm^−^^1^
α = 93.28 (3)°	*T* = 293 K
β = 90.67 (3)°	Block, yellow
γ = 113.47 (3)°	0.20 × 0.20 × 0.15 mm
*V* = 446.33 (15) Å^3^	

### Data collection

**Table:**

Bruker APEXII CCD diffractometer	2021 independent reflections
Radiation source: fine-focus sealed tube	1905 reflections with *I* > 2σ(*I*)
graphite	*R*_int_ = 0.009
φ and ω scans	θ_max_ = 28.3°, θ_min_ = 2.0°
Absorption correction: multi-scan (*SADABS*; Bruker, 2008)	*h* = −7→8
*T*_min_ = 0.852, *T*_max_ = 0.887	*k* = −8→8
2811 measured reflections	*l* = −13→13

### Refinement

**Table:**

Refinement on *F*^2^	Primary atom site location: structure-invariant direct methods
Least-squares matrix: full	Secondary atom site location: difference Fourier map
*R*[*F*^2^ > 2σ(*F*^2^)] = 0.027	Hydrogen site location: inferred from neighbouring sites
*wR*(*F*^2^) = 0.070	H atoms treated by a mixture of independent and constrained refinement
*S* = 1.07	*w* = 1/[σ^2^(*F*_o_^2^) + (0.030*P*)^2^ + 0.2201*P*] where *P* = (*F*_o_^2^ + 2*F*_c_^2^)/3
2021 reflections	(Δ/σ)_max_ = 0.002
146 parameters	Δρ_max_ = 0.27 e Å^−^^3^
0 restraints	Δρ_min_ = −0.25 e Å^−^^3^

### Special details

**Table:**

Geometry. All e.s.d.'s (except the e.s.d. in the dihedral angle between two l.s. planes) are estimated using the full covariance matrix. The cell e.s.d.'s are taken into account individually in the estimation of e.s.d.'s in distances, angles and torsion angles; correlations between e.s.d.'s in cell parameters are only used when they are defined by crystal symmetry. An approximate (isotropic) treatment of cell e.s.d.'s is used for estimating e.s.d.'s involving l.s. planes.
Refinement. Refinement of *F*^2^ against ALL reflections. The weighted *R*-factor *wR* and goodness of fit *S* are based on *F*^2^, conventional *R*-factors *R* are based on *F*, with *F* set to zero for negative *F*^2^. The threshold expression of *F*^2^ > σ(*F*^2^) is used only for calculating *R*-factors(gt) *etc*. and is not relevant to the choice of reflections for refinement. *R*-factors based on *F*^2^ are statistically about twice as large as those based on *F*, and *R*- factors based on ALL data will be even larger.

### Fractional atomic coordinates and isotropic or equivalent isotropic displacement parameters (Å^2^)

**Table:**

	*x*	*y*	*z*	*U*_iso_*/*U*_eq_	
Mn1	0.0000	0.5000	0.5000	0.02451 (10)	
O1	0.20420 (17)	0.69142 (18)	0.12393 (10)	0.0279 (2)	
O1W	−0.00060 (19)	0.21428 (19)	0.40159 (11)	0.0308 (2)	
H1WA	0.0974	0.1872	0.4325	0.046*	
O2	0.06195 (17)	0.64929 (19)	0.31264 (11)	0.0311 (3)	
O3	0.34009 (16)	0.63381 (18)	0.51888 (10)	0.0290 (2)	
O4	0.67461 (16)	0.86812 (18)	0.49213 (11)	0.0307 (3)	
C1	0.7033 (3)	0.7877 (3)	−0.12676 (17)	0.0377 (4)	
H1A	0.8140	0.8088	−0.1842	0.045*	
C2	0.7458 (3)	0.7881 (3)	0.00281 (16)	0.0327 (3)	
H2A	0.8845	0.8080	0.0326	0.039*	
C3	0.5793 (2)	0.7581 (2)	0.09021 (14)	0.0255 (3)	
C4	0.3735 (2)	0.7261 (2)	0.04195 (14)	0.0254 (3)	
C5	0.3284 (3)	0.7240 (3)	−0.08835 (15)	0.0332 (4)	
H5A	0.1893	0.7017	−0.1186	0.040*	
C6	0.4953 (3)	0.7559 (3)	−0.17236 (16)	0.0377 (4)	
H6A	0.4685	0.7562	−0.2601	0.045*	
C7	0.6108 (2)	0.7651 (2)	0.22644 (14)	0.0253 (3)	
H7A	0.7483	0.7901	0.2607	0.030*	
C8	0.4463 (2)	0.7363 (2)	0.30689 (13)	0.0218 (3)	
C9	0.2290 (2)	0.6896 (2)	0.25373 (14)	0.0230 (3)	
C10	0.4883 (2)	0.7473 (2)	0.45064 (14)	0.0223 (3)	
H1WB	−0.110 (4)	0.112 (4)	0.418 (2)	0.057 (7)*	

### Atomic displacement parameters (Å^2^)

**Table:**

	*U*^11^	*U*^22^	*U*^33^	*U*^12^	*U*^13^	*U*^23^
Mn1	0.01647 (15)	0.02948 (18)	0.02242 (17)	0.00348 (12)	0.00291 (11)	0.00327 (12)
O1	0.0219 (5)	0.0382 (6)	0.0210 (5)	0.0088 (4)	−0.0001 (4)	0.0052 (4)
O1W	0.0233 (5)	0.0326 (6)	0.0332 (6)	0.0077 (5)	0.0017 (4)	0.0022 (5)
O2	0.0191 (5)	0.0457 (7)	0.0274 (6)	0.0107 (5)	0.0038 (4)	0.0102 (5)
O3	0.0188 (5)	0.0397 (6)	0.0220 (5)	0.0042 (4)	0.0017 (4)	0.0066 (4)
O4	0.0185 (5)	0.0351 (6)	0.0297 (6)	0.0014 (4)	−0.0035 (4)	0.0037 (5)
C1	0.0473 (10)	0.0351 (9)	0.0282 (8)	0.0133 (7)	0.0157 (7)	0.0038 (7)
C2	0.0310 (8)	0.0342 (8)	0.0307 (8)	0.0104 (6)	0.0093 (6)	0.0030 (6)
C3	0.0263 (7)	0.0253 (7)	0.0223 (7)	0.0073 (6)	0.0042 (5)	0.0030 (5)
C4	0.0271 (7)	0.0236 (7)	0.0223 (7)	0.0066 (6)	0.0033 (5)	0.0030 (5)
C5	0.0389 (9)	0.0324 (8)	0.0242 (8)	0.0101 (7)	−0.0031 (6)	0.0016 (6)
C6	0.0558 (11)	0.0332 (8)	0.0199 (7)	0.0133 (8)	0.0046 (7)	0.0020 (6)
C7	0.0202 (6)	0.0277 (7)	0.0257 (7)	0.0072 (5)	0.0007 (5)	0.0020 (6)
C8	0.0186 (6)	0.0237 (7)	0.0210 (7)	0.0060 (5)	0.0001 (5)	0.0022 (5)
C9	0.0217 (7)	0.0241 (7)	0.0213 (7)	0.0066 (5)	0.0009 (5)	0.0049 (5)
C10	0.0174 (6)	0.0258 (7)	0.0224 (7)	0.0074 (5)	0.0000 (5)	0.0016 (5)

### Geometric parameters (Å, °)

**Table:**

Mn1—O3	2.0931 (12)	C1—C6	1.395 (3)
Mn1—O3^i^	2.0931 (12)	C1—H1A	0.9300
Mn1—O1W	2.1884 (13)	C2—C3	1.407 (2)
Mn1—O1W^i^	2.1884 (13)	C2—H2A	0.9300
Mn1—O2^i^	2.2315 (13)	C3—C4	1.389 (2)
Mn1—O2	2.2315 (13)	C3—C7	1.429 (2)
O1—C9	1.3626 (17)	C4—C5	1.386 (2)
O1—C4	1.3802 (18)	C5—C6	1.384 (3)
O1W—H1WA	0.8200	C5—H5A	0.9300
O1W—H1WB	0.82 (3)	C6—H6A	0.9300
O2—C9	1.2225 (18)	C7—C8	1.351 (2)
O3—C10	1.2549 (18)	C7—H7A	0.9300
O4—C10	1.2509 (17)	C8—C9	1.4555 (19)
C1—C2	1.377 (2)	C8—C10	1.5133 (19)
			
O3—Mn1—O3^i^	180.0	C3—C2—H2A	120.0
O3—Mn1—O1W	91.96 (5)	C4—C3—C2	118.33 (14)
O3^i^—Mn1—O1W	88.04 (5)	C4—C3—C7	117.86 (14)
O3—Mn1—O1W^i^	88.04 (5)	C2—C3—C7	123.78 (14)
O3^i^—Mn1—O1W^i^	91.96 (5)	O1—C4—C5	117.46 (14)
O1W—Mn1—O1W^i^	180.00 (6)	O1—C4—C3	120.28 (13)
O3—Mn1—O2^i^	97.12 (5)	C5—C4—C3	122.25 (15)
O3^i^—Mn1—O2^i^	82.88 (5)	C4—C5—C6	118.39 (16)
O1W—Mn1—O2^i^	91.16 (5)	C4—C5—H5A	120.8
O1W^i^—Mn1—O2^i^	88.84 (5)	C6—C5—H5A	120.8
O3—Mn1—O2	82.88 (5)	C5—C6—C1	120.65 (16)
O3^i^—Mn1—O2	97.12 (5)	C5—C6—H6A	119.7
O1W—Mn1—O2	88.84 (5)	C1—C6—H6A	119.7
O1W^i^—Mn1—O2	91.16 (5)	C8—C7—C3	121.79 (14)
O2^i^—Mn1—O2	180.0	C8—C7—H7A	119.1
C9—O1—C4	122.73 (12)	C3—C7—H7A	119.1
Mn1—O1W—H1WA	109.5	C7—C8—C9	119.37 (13)
Mn1—O1W—H1WB	110.8 (17)	C7—C8—C10	119.84 (13)
H1WA—O1W—H1WB	102.0	C9—C8—C10	120.77 (12)
C9—O2—Mn1	124.45 (10)	O2—C9—O1	114.86 (13)
C10—O3—Mn1	133.91 (10)	O2—C9—C8	127.35 (14)
C2—C1—C6	120.41 (16)	O1—C9—C8	117.78 (13)
C2—C1—H1A	119.8	O4—C10—O3	124.70 (14)
C6—C1—H1A	119.8	O4—C10—C8	116.15 (13)
C1—C2—C3	119.96 (16)	O3—C10—C8	119.13 (12)
C1—C2—H2A	120.0		
			
O3—Mn1—O2—C9	25.72 (12)	C2—C1—C6—C5	−0.1 (3)
O3^i^—Mn1—O2—C9	−154.28 (12)	C4—C3—C7—C8	1.7 (2)
O1W—Mn1—O2—C9	−66.40 (13)	C2—C3—C7—C8	179.71 (15)
O1W^i^—Mn1—O2—C9	113.60 (13)	C3—C7—C8—C9	2.2 (2)
O1W—Mn1—O3—C10	90.62 (15)	C3—C7—C8—C10	−179.37 (13)
O1W^i^—Mn1—O3—C10	−89.38 (15)	Mn1—O2—C9—O1	152.34 (10)
O2^i^—Mn1—O3—C10	−177.97 (14)	Mn1—O2—C9—C8	−28.2 (2)
O2—Mn1—O3—C10	2.03 (14)	C4—O1—C9—O2	−177.49 (13)
C6—C1—C2—C3	−0.6 (3)	C4—O1—C9—C8	3.0 (2)
C1—C2—C3—C4	0.8 (2)	C7—C8—C9—O2	176.00 (15)
C1—C2—C3—C7	−177.20 (15)	C10—C8—C9—O2	−2.4 (2)
C9—O1—C4—C5	179.93 (14)	C7—C8—C9—O1	−4.5 (2)
C9—O1—C4—C3	1.0 (2)	C10—C8—C9—O1	177.09 (12)
C2—C3—C4—O1	178.54 (13)	Mn1—O3—C10—O4	155.33 (12)
C7—C3—C4—O1	−3.3 (2)	Mn1—O3—C10—C8	−26.6 (2)
C2—C3—C4—C5	−0.4 (2)	C7—C8—C10—O4	31.1 (2)
C7—C3—C4—C5	177.76 (14)	C9—C8—C10—O4	−150.50 (14)
O1—C4—C5—C6	−179.23 (14)	C7—C8—C10—O3	−147.10 (15)
C3—C4—C5—C6	−0.3 (2)	C9—C8—C10—O3	31.3 (2)
C4—C5—C6—C1	0.5 (3)		

Symmetry codes: (i) −*x*, −*y*+1, −*z*+1.

### Hydrogen-bond geometry (Å, °)

**Table:**

*D*—H···*A*	*D*—H	H···*A*	*D*···*A*	*D*—H···*A*
O1W—H1WA···O4^ii^	0.82	1.89	2.7113 (17)	178
O1W—H1WB···O4^iii^	0.82 (3)	1.95 (3)	2.755 (2)	167 (2)

Symmetry codes: (ii) −*x*+1, −*y*+1, −*z*+1; (iii) *x*−1, *y*−1, *z*.

## References

[bb1] Bruker (2008). *APEX2*, *SAINT* and *SADABS* Bruker AXS Inc., Madison, Wisconsin, USA.

[bb2] Gao, Q., Jiang, F. L., Wu, M. Y., Huang, Y. G., Wei, W. & Hong, M. C. (2010). *Cryst. Growth Des.*, **10**, 184–190.

[bb3] Hazra, A. J., Ali, Z. M., Shah, F. A., Zardari, L. A. & Khuhawar, M. Y. (2011). *Chem. Commun.* **47**, 538–540.

[bb4] Hu, J. S., Shang, Y. J., Yao, X. Q., Qin, L., Li, Y. Z., Guo, Z. J., Zheng, H. G. & Xue, Z. L. (2010). *Cryst. Growth Des.* **10**, 4135–4142.

[bb5] Kuschel, A., Luka, M., Wessig, M., Drescher, M., Fonin, M., Kiliani, G. & Polarz, S. (2010). *Adv. Funct. Mater.* **20**, 1133–1143.

[bb6] Sheldrick, G. M. (2008). *Acta Cryst.* A**64**, 112–122. 10.1107/S0108767307043930 18156677

[bb7] Yang, Q., Zhao, J. P., Hu, B. W., Zhang, X. F. & Bu, X. H. (2010). *Inorg. Chem.* **49**, 3746–3751. 10.1021/ic9021257 20329767

